# The effect of long-term dehydration and subsequent rehydration on markers of inflammation, oxidative stress and apoptosis in the camel kidney

**DOI:** 10.1186/s12917-020-02628-5

**Published:** 2020-11-23

**Authors:** Mahmoud A. Ali, Hassan Abu Damir, Osman M. Ali, Naheed Amir, Saeed Tariq, Michael P. Greenwood, Panjiao Lin, Benjamin Gillard, David Murphy, Abdu Adem

**Affiliations:** 1 Department of Pharmacology, CollegeofMedicine&HealthSciences, United Arab Emirates University, Al- Ain, United Arab Emirates; 2 College of Medicine and Public Health, Flinders University, Adelaide, Australia; 3 Department of Anatomy, CollegeofMedicine&HealthSciences, Emirates University, Al-Ain, United Arab Emirates; 4 Molecular Neuroendocrinology Research Group, Bristol Medical School, Translational Health Sciences, University of Bristol, Dorothy Hodgkin Building, Bristol, BS13NY UK; 5 Department of Pharmacology, College of Medicine and Health Sciences, Khalifa University, P.O.Box 127788, Abu Dhabi, UAE

**Keywords:** Dromedary camels, Dehydration/rehydration, Kidney cortex/medulla, Gene expression, Oxidative stress and apoptosis, Pro-inflammatory markers

## Abstract

**Background:**

Dehydration has deleterious effects in many species, but camels tolerate long periods of water deprivation without serious health compromise. The kidney plays crucial role in water conservation, however, some reports point to elevated kidney function tests in dehydrated camels. In this work, we investigated the effects of dehydration and rehydration on kidney cortex and medulla with respect to pro-inflammatory markers, oxidative stress and apoptosis along with corresponding gene expression.

**Results:**

The cytokines IL-1β and IL-18 levels were significantly elevated in the kidney cortex of dehydrated camel, possibly expressed by tubular epithelium, podocytes and/or mesangial cells. Elevation of IL-18 persisted after rehydration. Dehydration induced oxidative stress in kidney cortex evident by significant increases in MDA and GSH, but significant decreases in SOD and CAT. In the medulla, CAT decreased significantly, but MDA, GSH and SOD levels were not affected. Rehydration abolished the oxidative stress. In parallel with the increased levels of MDA, we observed increased levels of *PTGS1* mRNA, in MDA synthesis pathway. *GCLC* mRNA expression level, involved in GSH synthesis, was upregulated in kidney cortex by rehydration. However, both *SOD1* and *SOD3* mRNA levels dropped, in parallel with SOD activity, in the cortex by dehydration. There were significant increases in caspases 3 and 9, p53 and PARP1, indicating apoptosis was triggered by intrinsic pathway*.* Expression of *BCL2l1* mRNA levels, encoding for BCL-xL, was down regulated by dehydration in cortex. *CASP3* expression level increased significantly in medulla by dehydration and continued after rehydration whereas *TP53* expression increased in cortex by rehydration. Changes in caspase 8 and TNF-α were negligible to instigate extrinsic apoptotic trail. Generally, apoptotic markers were extremely variable after rehydration indicating that animals did not fully recover within three days.

**Conclusions:**

Dehydration causes oxidative stress in kidney cortex and apoptosis in cortex and medulla. Kidney cortex and medulla were not homogeneous in all parameters investigated indicating different response to dehydration/rehydration. Some changes in tested parameters directly correlate with alteration in steady-state mRNA levels.

## Background

The nephrons, located mostly in kidney cortex, are involved in the ultrafiltration of arterial blood, reabsorption of water, electrolytes and smaller molecules, whereas the medullary components of kidney, with the exception of the Henle loops and collecting ducts, are principally engaged in urine excretion. A number of hormones, peptides, receptors, aquaporins and solute carrier proteins work in harmony within the kidney tubules to maintain water and electrolyte balance [36, 28, 35]. The camel kidney, unlike human, horse and cattle, is bean-shaped with a smooth surface and prominent medulla compared to cortex, with a volume ratio of 4:1 [1]. The dromedary camel nephron is similar to that of other species in terms of physiology, histology, morphometry and electron microscopy,however, it is peculiarly adapted to achieve the increased water reabsorption and urine concentration necessary for life in the arid desert environment [47, 55].

However, it is worth noting that small vesicular membrane-bound bodies have been reported in the dromedary camel kidney, which may be engaged in water and solute regulation [8, 9, 19]. Long-term dehydration can cause oxidative stress with increased reactive oxygen species (ROS), pro-inflammatory interleukins such as Interleukin 1β (IL-1β), Interleukin 6 (IL-6), Interleukin 18 (IL-18) and Tumor Necrosis Factor α (TNF-α) and apoptotic markers [5]. Acute-injury of the kidney can result in DNA damage (with failure to repair), crowding of endoplasmic reticulum (ER) with an unfolded protein response (UPR), increased reactive oxygen species (ROS) and increased cellular apoptosis, amongst other effects [41, 46]**.** Recurrent acute or chronic dehydration has also been implicated in insidious kidney injury in human and laboratory animals, which ultimately advances into chronic renal disease [12, 26]. The recently emerged disease, Mesoamerican nephropathy, diagnosed in sugar-cane farmers in Central America, exemplifies this [44, 56]. On the other hand, desert animals display less severe responses to acute or chronic dehydrations [34, [33]. Although these animals undergo cellular stress, kidney apoptosis and tissue damage, consequences are less pronounced, which has been attributed to an acquired adaptive response to the harsh arid environment [13, 31]. For example, desert mice subjected to acute dehydration displayed decreased kidney matrix-turnover and limited apoptosis with normal creatinine and other kidney markers,however, there was more expression of genes encoding for aquaporin and solute carrier proteins [34, 33]. Long-term dehydration in the arid environment with its hot humid weather is very challenging to the dromedary camel [49] and [22]. Although, the camel kidney, like other desert mammals, is very efficient in water and electrolyte regulation [37], some reports point to high elevation in some kidney function tests, such as blood urea nitrogen, creatinine, sodium, osmolality and other relevant hormonal and peptides parameters [4, 2]. It is worth mentioning that oxidative stress, histopathological changes and apoptotic markers were studied in our recent publication on the stomach of dehydrated camels [6]. However, our current knowledge of the effects of dehydration and rehydration on the camel kidney is limited. It is not known whether dehydration causes acute or chronic, temporary or permanent, effects on the camel kidney, or whether the kidney is refractory to stress, resuming full functionality immediately after rehydration with no further consequences.

The aim of this work was to investigate the effects of long-term dehydration and rehydration on pro- inflammatory cytokine markers, oxidative stress biomarkers and apoptosis markers in both cortex and medulla of the camel kidney. The expression of genes encoding for proteins and enzymes involved in these processes was also investigated.

## Results

### Body weight

There was no significant difference between all groups at the start of the experiment [30]. The body weights (mean ± SEM) of the control group did not change significantly during the experimental period from (356.8 ± 10.3 to 364.1 ± 15 kg). The body weights of the dehydrated group decreased significantly at the end of the dehydration period (251.8 ± 10.1 vs 364.1 ± 15 kg) constituting 31% weight loss. Two hours after rehydration the body weights of the rehydrated camels increased by 17% (251.8 ± 10.3 vs 294.5 ± 13 kg) compared to the dehydrated body weight.

### Measurement of pro-inflammatory markers

**Fig. 1:**
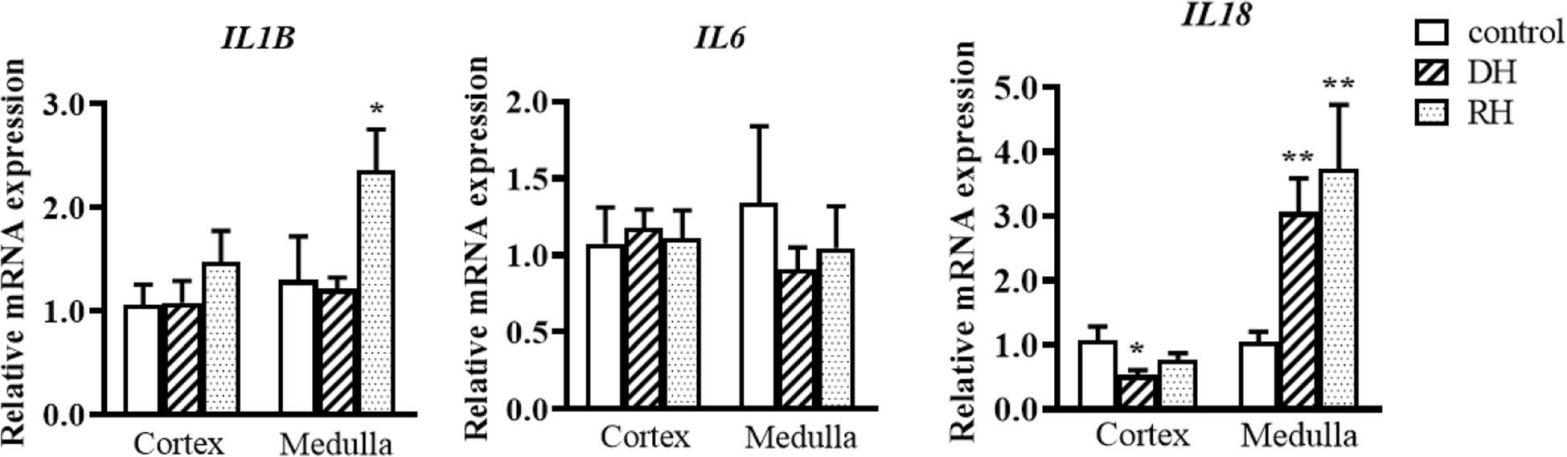
Relative mRNA expression of pro-inflammatory markers in kidney cortex and medulla of Control, Dehydrated (DH) and Rehydrated (RH) Camels (mean ± SEM). qPCR was performed to examine mRNA expression of IL-1β, IL-6 and IL-18. Significant difference from Control group denoted by *p* < 0.05: *, *p* < 0.01: **

ELISA was used to measure steady state levels of pro-inflammatory interleukin markers (IL-1β, IL-6, IL-18) in control, dehydrated and rehydrated camel kidney cortex and medulla Table 1. IL-1β in the dehydrated camel cortex displayed significantly higher values compared to rehydrated (*p* < 0.05) and control (*p* < 0.05) camels, whilst there was no significant difference between the rehydrated and control. No significant changes were apparent in kidney medulla. IL-6 levels did not change significantly comparing dehydrated, rehydrated and control groups in both cortex and medulla**.** IL-18 levels showed significant differences between dehydrated and control groups in the cortex. Moreover, the IL-18 levels in the cortex increased significantly after rehydration compared to the controls and dehydrated. However, the levels of IL-18 decreased significantly in the medulla of the dehydrated camels compared to the controls Table 1. At the steady-state mRNA level, qPCR revealed that IL-1β mRNA in the kidney medulla of rehydrated camels showed significantly higher expression level compared to control camels whereas no significant between the dehydrated and control values was observed Fig. 1. IL-6 values displayed no significant difference among the control and experimental groups in cortex and medulla. The mRNA expression level of IL-18 in the kidney cortex of dehydrated camel was significantly lower than the control values. Additionally, the IL-18 expression levels in the medulla increased significantly after dehydration and maintained until rehydration compared to the controls Fig. 1.

**Table 1:** Pro-inflammatory markers (IL-1β, IL-18 and IL-6) in renal cortex and medulla of Control Dehydrated and Rehydrated camels (mean ± SEM)

Parameter	Tissue type	Control	Dehydrated	Rehydrated
**IL-1β** pg/mg tissue	Cortex	20.7 ± 1.2	23.8 ± 0.6*	20.5 ± 1.5†
	Medulla	18.9 ± 1.3	18.5 ± 0.8	21.4 ± 1.5
**IL-6** pg/mg tissue	Cortex	15.1 ± 1.1	16.5 ± 1.4	18.7 ± 2.3
	Medulla	7.7 ± 1.0	6.2 ± 0.3	8.8 ± 2.5
**IL-18** pg/mg tissue	Cortex Medulla	8.17 ± 0.26 19.54 ± 1.71	12.19 ± 0.28*** 15.03 ± 0.90*	15.41 ± 0.55***†† 15.54 ± 1.18

Significant difference from Control group is denoted by *p < 0.05, ***p < 0.001 and from Dehydrated group by ^†^*p* < 0.05, ^††^*p* < 0.01

### Measurement of oxidative stress biomarkers

Oxidative stress biomarkers Malondialdehyde (MDA), Total Glutathione (GSH), Superoxide dismutase (SOD), and Catalase (CAT) were quantified Table 2. The concentration of MDA in the camel kidney cortex was significantly higher in dehydrated camels compared to rehydrated and control camels but no significant difference between the rehydrated and control groups was observed. The kidney medulla displayed no significant differences in MDA between the three groups. GSH level showed a significant increase in the kidney cortex of dehydrated camels compared to rehydrated and control groups, which were comparable.

**Table 2:** Oxidative Stress Biomarkers in kidney Cortex and Medulla of Control, Dehydrated and Rehydrated camels (mean ± SEM)

Parameter	Tissue type	Control	Dehydrated	Rehydrated
MDA µM	Cortex Medulla	3.28 ± 0.49 1.95 ± 0.54	5.03 ± 0.47* 2.20 ± 0.40	3.71 ± 0.33† 2.27 ± 0.19
GSH (µM)	Cortex	4.96 ± 0.34	6.79 ± 0.35**	5.15 ± 0.29 †
	Medulla	5.88 ± 0.33	5.87 ± 0.36	6.04 ± 0.96
SOD U/mg	Cortex Medulla	572.70 ± 43.37 356.04 ± 46.01	398.47 ± 55.01* 377.78 ± 26.14	605.48 ± 29.73† 288.42 ± 72.32
CAT nmol/min/mg	Cortex Medulla	104.78 ± 6.47 125.66 ± 13.49	56.93 ± 6.86*** 77.75 ± 13.34*	100.83 ± 4.44† 123.31 ± 10.51†

Significant difference from control group is denoted by *p < 0.05, **p < 0.01, ***p < 0.001 and from Dehydrated group by ^†^*p* < 0.05.

**Fig. 2:**
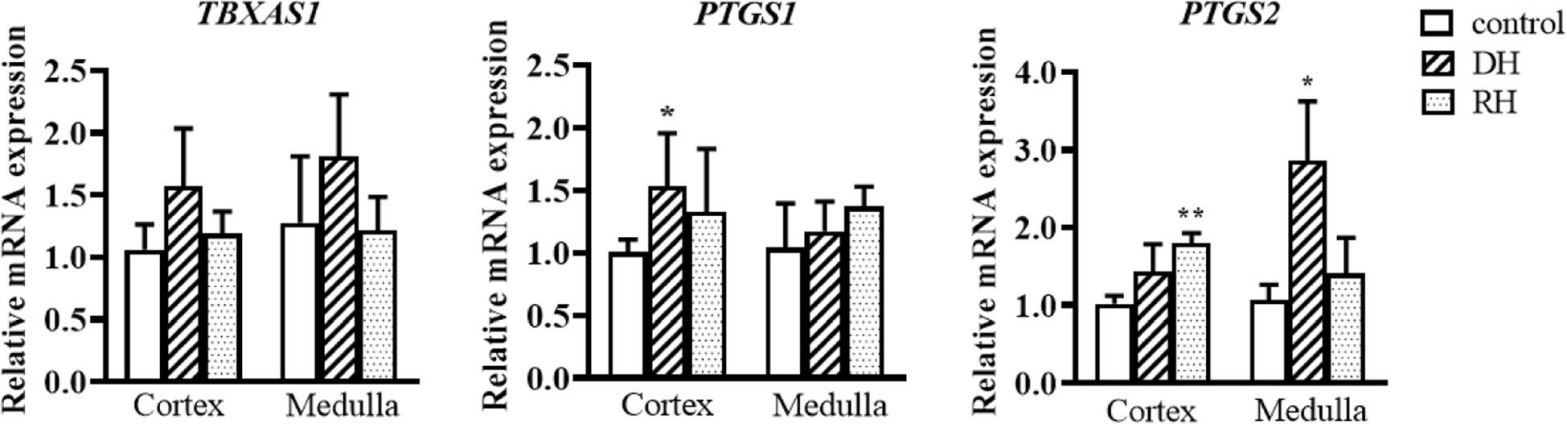
Relative mRNA expression of MDA synthesis-related genes in kidney cortex and medulla of Control, Dehydrated (DH) and Rehydrated (RH) Camels (mean ± SEM). qPCR was performed to examine mRNA expression of *TBXAS1, PTGS1* and *PTGS2*. Significant difference from Control group denoted by *p* < 0.05: *, *p* < 0.01: **

**Fig. 3:**
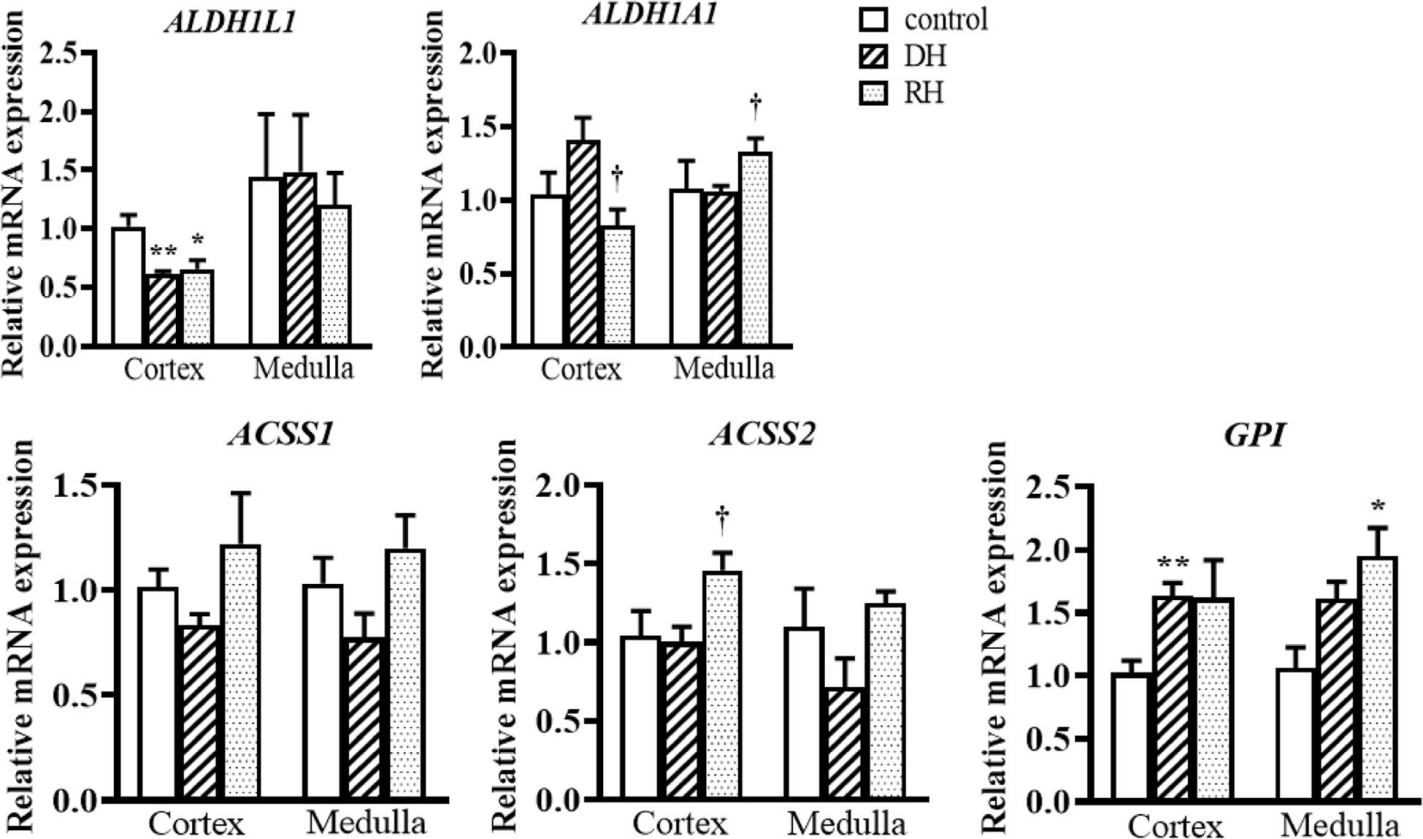
Relative mRNA expression of MDA catabolism-related genes in kidney cortex and medulla of Control, Dehydrated (DH) and Rehydrated (RH) Camels (mean ± SEM). qPCR was performed to examine mRNA expression of *ALDH1L1, ALDH1A1, ACSS1, ACSS2 and GPI*. Significant difference from Control group denoted by *p* < 0.05: *, *p* < 0.01: ** and from Dehydrated group by, *p* < 0.05: †

**Fig. 4:**
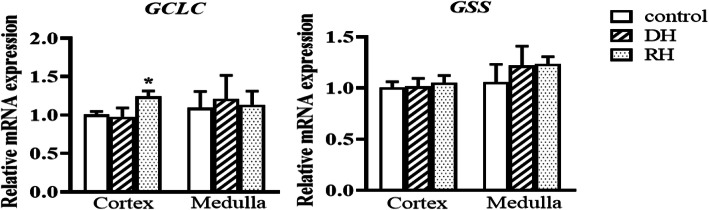
Relative mRNA expression of GSH synthesis-related genes in kidney cortex and medulla of Control, Dehydrated (DH) and Rehydrated (RH) Camels (mean ± SEM). qPCR was performed to examine mRNA expression of *GCLC and GSS*. Significant difference from Control group denoted by *p* < 0.05: *

**Fig. 5:**
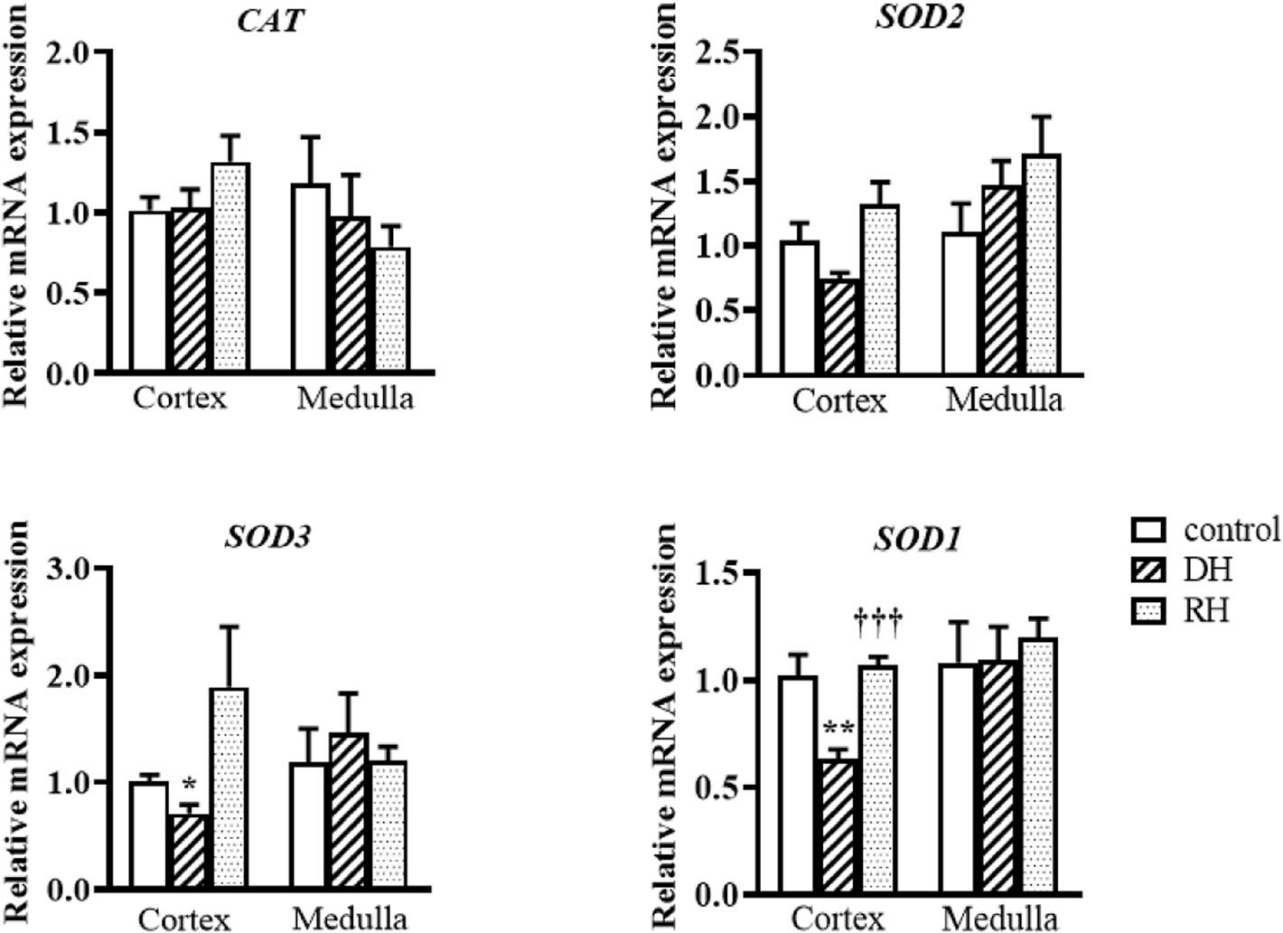
Relative mRNA expression of oxidative stress markers in kidney cortex and medulla of Control, Dehydrated (DH) and Rehydrated (RH) Camels (mean ± SEM). qPCR was performed to examine mRNA expression of *CAT, SOD1, SOD2 and SOD3*. Significant difference from Control group denoted by *p* < 0.05: *, *p* < 0.01: ** and from Dehydrated group by *p* < 0.001: †††

The activity of SOD is significantly decreased in the cortex of dehydrated camels compared to the rehydrated and control groups, which were not statistically different. The GSH concentration and SOD activity in the medulla of the three groups revealed no significant difference. CAT activity was significantly lower in the cortex (p < 0.001) and medulla (p < 0.05) of dehydrated compared to the rehydrated and control groups. However, CAT values in rehydrated and control camels' cortices as well as medullae exhibited no significant difference Table 2. Steady-state mRNA expression levels of genes encoding oxidative stress biomarkers (CAT and SOD1-3) and genes involved in the metabolisms of other oxidative stress markers (MDA and GSH) were quantified by qPCR Figs 2, 3, 4 and 5. The expression of steady state mRNAs that encode enzymes responsible for MDA synthesis (the TBXAS1,PTGS1 and PTGS2 genes) are presented in Fig. 2. No significant differences were observed for TBXAS1 among the control and experimental values in both cortex and medulla. For PTGS1, the expression level in cortex increased significantly during dehydration in comparison with the controls. Moreover, the *PTGS2* mRNA expression level was significantly higher in the kidney cortex of rehydrated camels than the control camels.

However, in the kidney medulla, the expression level of PTGS2 showed significant increase during dehydration ACSS2 expression level significantly increased after being subjected to rehydration in the kidney cortex. The expression of GPI significantly increased during dehydration in cortex and during rehydration in medulla compared to the control groups Fig. 3. We observed different patterns for the expression of two genes involved in GSH synthesis Fig. 4. GCLC transcript levels displayed a significant up-regulation in kidney cortex during rehydration in comparison with the control groups. GSS mRNA expression levels showed no significant difference between the three groups in both cortex and medulla Fig. 4.

In terms of the expression level of the oxidative stress markers, Fig. 5 illustrates that although no significant differences were observed for the expression levels of CAT and SOD2 mRNAs in both cortex and medulla between the three groups, SOD1 and SOD3 transcript expression levels in the kidney cortex during the dehydration stage were down-regulated significantly when compared with the control camels. Compared with the dehydrated group, the expression level of SOD 1 mRNAs in the cortex of the rehydrated group was significantly increased to attain the control level Fig. 5.

### Measurement of apoptotic markers

**Fig. 6:**
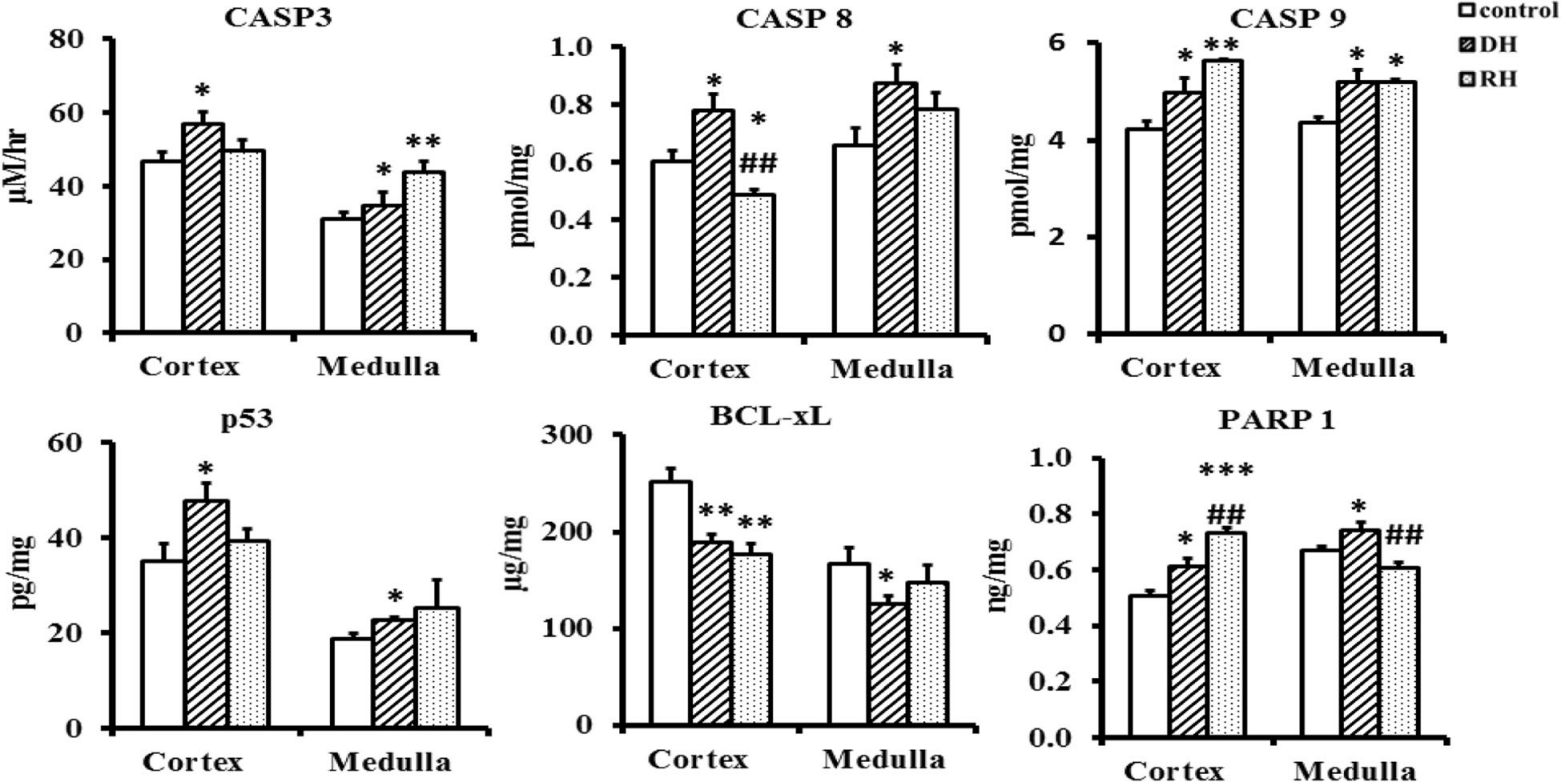
Apoptotic markers in kidney cortex and medulla of Control**,** Dehydrated (DH) and Rehydrated (RH) Camels (mean ± SEM). Significant difference from Control group denoted by *p < 0.05, **p < 0.01, ***p < 0.001 and from Dehydrated group by, **#p** < 0.05, **##p** < 0.01

ELISA quantification of steady-state levels of apoptotic protein markers; Caspase 3 (CASP3), Caspase8 (CASP8), Caspase 9 (CASP9), Tumor protein 53 (p53), B-cell lymphoma-extralarge (Bcl-xL), Poly ADP-ribose Polymerase (PARP1) in camel kidney cortex and medulla of control, dehydrated and rehydrated camel is presented in Fig. 6. Caspase 3, p53 and Bcl-xL are more expressed in cortex,PARP1 is more expressed in medulla, while caspases 8 and 9 are equally expressed in cortex and medulla. Caspases (3, 8, 9), p53 and PARP1 significantly increased, whilst BCL-xL significantly decreased in response to dehydration in both cortex and medulla. During the rehydration period, the levels of caspase 3 and p53 dropped in cortex to reach control values, whilst the medullary values continued increasing only in the caspase 3. Caspase 9 and PARP1 continued increasing following rehydration in cortex, whilst in medulla, only PARP1 dropped significantly (*p* < 0.001). Bcl-xL continued significant increase (*p* < 0.05) in caspase 8 as a result of dehydration in both cortex and medulla, and values decreased to control values or significantly below in medulla and cortex respectively following rehydration Fig. 6.

**Fig. 7:**
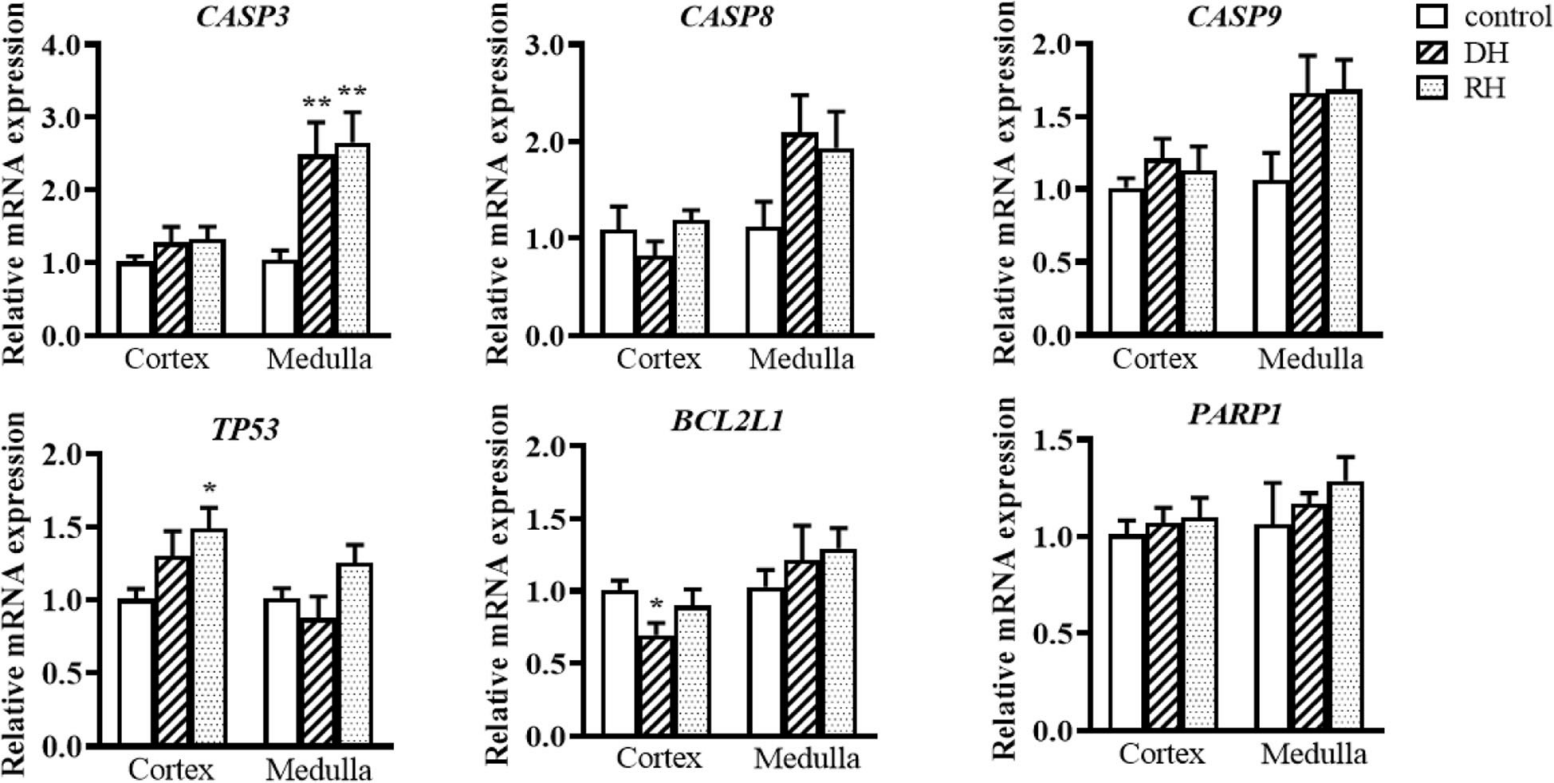
Relative mRNA expression of mRNAs encoding apoptotic markers in kidney cortex and medulla of Control, Dehydrated (DH) and Rehydrated (RH) camels (mean ± SEM). qPCR was performed to examine mRNA expression of *CASP3, CAS8, CASP9, TP53, BCL2L1 and PARP1*. Significant difference from Control groups denoted by *p* < 0.05: *, *p* < 0.01: **

The steady-state expression levels of mRNAs encoding apoptotic markers (*CASP3, CASP8, CASP9, TP53, BCL2L1* [encoding Bcl-xL] and *PARP1*) are presented in Fig. 7. The expression of *CASP8, CASP9* and *PARP1* were not significantly different between the three groups in both cortex and medulla. For *CASP3*, its expression dropping in cortex (*p* < 0.01) of rehydrated camels but increased in medulla to reach control levels. Caspase 8 values were very low in kidney compared to organs such as abomasum (results not shown). However, there is slight but significant increase (*p* < 0.05) in caspase 8 as a result of dehydration in both cortex and medulla, and values decreased to control values or significantly below in medulla and cortex respectively following rehydration Fig. 6.

The steady-state expression levels of mRNAs encoding apoptotic markers (*CASP3, CASP8, CASP9, TP53, BCL2L1* [encoding Bcl-xL] and *PARP1*) are presented in Fig. 7. The expression of *CASP8, CASP9* and *PARP1* were not significantly different between the three groups in both cortex and medulla. For *CASP3*, its expression level in kidney medulla significantly increased during dehydration and continued increasing during the rehydration period. *TP53* expression significantly increased in the cortex of the rehydrated camels compared to the control camels. The expression of the mRNA encoding BCL-xl (*BCL2L1*) was down regulated by dehydration in kidney cortex.

## Discussion

Long-term dehydration in the dromedary camel is accompanied by profound physiological, biochemical and hematological changes [25, 48, 5]. In a previous paper, we reported some histopathological lesions in gastric mucosa,elevated oxidative stress markers and increased apoptosis accompanied changes in the levels of neuropeptide hormones, prostaglandin E2 and H^+^/K^+^ ATPase to modulate the body activities towards water economy [6]. Generally, the kidneys of desert animals such as desert rodents displayed less severe inflammatory, oxidative and apoptotic responses to dehydration [35] compared to non-desert species where long-term dehydration or intermittent dehydration can cause severe kidney damage. The camel, being adapted to the harsh arid environment, can live without water for long periods similar to other desert species [13]. Therefore, it is interesting to investigate the extent of inflammation, oxidative stress and apoptosis when the camel kidney is subjected to long-term dehydration and subsequent rehydration and if these processes are directly correlated to alteration in steady-state m-RNA and if they have any effects on renal function. In this report, we presented new data pertinent to effects of dehydration/rehydration on kidney cortex and medulla. IL-18 and IL-1β are pro-inflammatory cytokines primarily produced by activated macrophages [23, 58] and both overlap in many functions but differ in signaling pathways with IL-1β being highly involved in pyrexia [27]. IL-18 is also expressed in many other cells, including the renal tubular epithelium, podocytes and mesangial cells [51, 57], and is considered an acute marker of tubular necrosis [42, 43]. IL-18 and IL-12 synergistically stimulate the production of interferon gamma (IFNγ) by T lymphocytes and NK cells altering the transcription of more than 30 genes to elicit a number of inflammatory and autoimmune diseases [34, 53]. The increased IL-18 values seen in kidney cortex of dehydrated and rehydrated camels might have played crucial roles in gene expression and lesions particularly seen in podocytes, mesangial cells, capillaries, proximal and distal convoluted tubules, whereas the medullary collecting ducts and parenchyma seemed to be refractory.

The discriminate rise of IL-18 in cortex and drop in medulla of dehydrated and rehydrated camels compared to controls might indicate that the kidney cortical cells independent of macrophage might be the source of IL-18 productions similar to that observed in obstructive kidney injury [21]. The rise of IL-1β in kidney cortex of dehydrated camels is not surprising as it is involved in inflammation, apoptosis and other activities. In contrast to the cortex, IL-1β levels in medulla of dehydrated camels were not changed indicating little or no production of IL-1β at this site. No changes seen in IL-6 protein levels in either cortex or medulla. Similarly, no changes observed in steady state *IL-6* mRNA levels. However, there was no direct relationship between IL-1β and IL-18 protein abundance and the steady-state expression levels of the corresponding mRNAs. Indeed, whilst IL-18 protein increased because of dehydration in the cortex, the mRNA level decreased. Similarly, in the medulla, whilst dehydration caused IL-18 protein levels to go down, mRNA abundance went up.

ROS molecules (superoxides, peroxides, triple oxide radicals, oxidized thiol) are normally produced because of metabolic processes in the body. However, enzymatic (SOD, CAT) and non-enzymatic (GSH, free thiol, Vitamins A, C and E) antioxidants scavenge these to maintain a balanced redox status in the body [54, 6]. On the other hand, disease conditions, chemicals toxins, strenuous exercise and dehydration can instigate over production of ROS, which rapidly proceed to form hydroxyl radical (OH) and peroxynitrite oxidant (ONO2) causing lipid peroxidation, proteins oxidation and DNA damage [15, 52]. It is well known that the carbon–carbon double bond of polyunsaturated fatty acids are the target of lipid peroxidation by free radical and cytochrome oxidase with MDA and 4-HNE molecules as byproducts [7]. The high levels of the lipid oxidation marker, MDA, and antioxidant molecule GSH, and the depletion of SOD and CAT enzymes in the kidney cortex of dehydrated camels were clear signals of tissue oxidative stress. On the other hand, the kidney medulla seemed to be refractory to ROS as there was no changes in MDA, GSH, and SOD levels of dehydrated camels compared to controls. We also examined the steady-state levels of mRNAs that encode the enzymes responsible for MDA and GSH metabolism, and of the mRNAs that encode SOD and CAT. Interestingly, in addition to the increased levels of MDA in the cortex due to dehydration, we observed increased levels of *PTGS1* mRNA, which is part of the pathway responsible for MDA synthesis. At the same time, we saw a decrease in the level of the mRNA encoding the MDA catabolic enzyme ALDH1L1. Similarly, levels of the *GCLC* mRNA, whilst not increased by dehydration in the cortex, were increased by rehydration, in parallel with the levels of GSH. Whilst *CAT* mRNA abundance did not change in the cortex as a consequence of dehydration, both *SOD1* and *SOD3* mRNA levels dropped, in parallel with SOD activity. Caspase enzymes, through intrinsic and extrinsic pathways [50, 59], induce apoptosis. The intrinsic pathway seems to be involved in the kidney cortex and medulla of dehydrated camels where caspases 9, 3 and p53 showed significant elevation. Normally**,** p53 concentration inside the cell is very low due to continuous turnover, but its level may become stabilized by stress to up-regulate various genes involved in apoptosis and other activities [40]. The intrinsic pathway of apoptosis seems to be dominant in the kidney of the dehydrated camel. The stress of long-term dehydration may induce DNA damage in cortical cells that will led to a significant elevation of p53 protein with activation of the pro-apoptotic factors, BAX and BAK via PUMA protein [16, 40]. These pro-apoptotic proteins lead to mitophagy, and promote mitochondrial membranes to release cytochrome c, apoptosis inducing factor (AIF). These, in turn, form complexes in the cytosol and activate caspase 9 and hence caspase 3, leading, ultimately, to cell execution [10, 40]. BCL-xl is an anti-apoptotic protein, encoded by the *BCL2L1* gene [11]. Its main function is to protect the mitochondrial membranes from pro-apoptotic proteins to form pores and facilitate the release of cytochrome c [18, 40]. BCL-xl also functions in the regulation of ATP synthesis, autophagy and cell mitosis [38]. There was a significant decrease of BCL-xl concentration in both kidney cortex and medulla of dehydrated camels, suggesting that apoptosis might be activated [20, 38]. PARP1 is usually engaged in DNA repair however, the enzyme remains active even if damage could not be amended, a process that leads to NAD^+^ depletion and ATP overconsumption to escalate a cell energy crisis [3, 39]. This leads to collapse of mitochondrial membrane potential, passage of apoptotic inducing factor, fragmentation of DNA and condensation of chromatin material [17, 29]. We report increased PARP activity in both cortex and medulla of dehydrated camels with a further elevation of PARP1 activity in the cortex 72-h post rehydration. However, it is not clear whether the rise of PARP1 plays a potential role in the programed cell death reported in the kidney of the dehydrated camels. It is worth mentioning that PARP1 activity in the medulla returned to normal along with ATP synthase activity, which might point to NAD^+^/ATP restoration early in that part of kidney. Supporting data for this notion will be published in another manuscript.

Quantification of steady-state mRNA levels suggested that the activation of apoptosis might be regulated, at least in part, at this level. Thus, *CASP3* mRNA levels are induced by dehydration in the medulla in parallel with the protein, whereas *BCL2L1* mRNA levels decrease in the cortex, along with the corresponding BCL-xL protein.

We were interested to ask whether alterations in steady-state mRNA levels were responsible for the inflammatory, oxidative stress and apoptotic changes observed as a consequence of dehydration and rehydration. Some direct correlations were observed, but in the main, our results suggest that, under these stressful conditions, there is no necessary direct relationship between steady-state mRNA levels and the abundance or activity of the corresponding protein. In these cases, regulation is presumably being effected downstream of steady-state mRNA level, perhaps, for different proteins, at different levels; for example, translation rate, protein compartmentalization, transport, translocation, secretion, post-translational modification, protein stability and activity-dependent turnover [33].

### Some limitations of our study deserve mention

Although we standardized the samples as much as possible, some variation is inevitable with the use non-model organisms such as the dromedary camel. We would have preferred direct measurements of body weight over derived values based on a number of length measurements each with their own potential for error. Despite best efforts to minimize the time between slaughter and tissue harvesting, some samples were exposed longer at ambient temperature before freezing, potentially affecting their quality. Further, the samples were kept frozen for a number of years at -80C to maintain integrity, but some degradation might have occurred. We noted differences in age and weight of our camels, with some animals being older and heavier than others, which could affect the response to dehydration. Physical differences were also observed, with animals differing in coat colours, for example. Crossbreeding to give rise to animals with specific characteristics is a common practice in the region, and colour is often used to monitor this. This could result in genetic variations in the kidney. Studies based on non-model species are expected to exhibit considerable biological variation, which engenders uncertainty with regards to physiological and genetic measurements regardless of the technology applied. These challenges should be understood as an unavoidable limitation of doing research with non-model species. Nonetheless, we are confident that the variability shown in these experiments is a consequence of biological differences rather than technical bias.

## Conclusions

Long-term dehydration and subsequent rehydration provoke profound changes in the kidney. We have documented increases in pro-inflammatory cytokines, oxidative stress and apoptosis. In addition to some gene expression that support these changes. However, kidney cortex and medulla often differ in their responses to dehydration and short-term rehydration.

## Methods

### Chemicals, kits and antibodies

Sodium deoxycholate, total Glutathione (GSH) enzyme kinetic assay kit, 3,3′,5,5′-Tetramethylbenzidine (TMB), and all other chemicals, if not specified were purchased from Sigma–Aldrich Chemical Company (Sigma Chemical Co., St. Louis, MO, USA). Complete protease inhibitor cocktail was purchased from Thermo Fisher Scientific Inc. (MA USA, 02,451). Catalase (CAT), Superoxide dismutase (SOD), assay kits were purchased from (Cayman Chemical Company, Ann Arbor, MI, USA). Bcl-xL, p53 ELISA kits were purchased from R&D Systems (MN, USA). Assay kits for camel ELISA and for IL-1β, IL-6, IL-18, TNF-α, Caspase 8, 9, PARP1, were purchased from MyBiosource, Inc. (CA 92,195–3308, USA). Caspase 3 assay kits were purchased from Promega Corporation (Madison, WI 53,711, USA). Malondialdehyde (MDA) Assay kits were purchased from Northwest Life Science Specialties, (WA, USA).

### Animals

Nineteen healthy male one-humped camels, 4–5 years old with body weight range of 276–416 kg, were used for this study. Animals were purchased from a local camel trading company (ALBWADRY CAMEL TRADING COMPANY). After a short adjustment period, the camels were divided randomly into three groups; Control group (n = 5), Dehydrated group (n = 8), and rehydrated group (n = 6). The camels were visited daily by an experienced Veterinarian to ensure their wellbeing. The control group were supplied with feed and water ad libitum for the whole experimental period. The dehydrated group were provided with feed ad libitum but without water for 20 days, while the rehydrated group was provided with feed ad libitum, restricted of water for 20 days and thereafter allowed free access to water for 72 h. The animals were kept in a shaded corral during the months of (April and May, 40–50 °C) in the Eastern Region of the United Arab Emirates. The camels were maintained on alfalfa green grass and dry hay. At the end of the experimental period, camels were sacrificed in the central abattoir for human consumption. Camel kidney samples were collected from all camels.

### Preparation of kidney homogenate

Kidney homogenate was prepared as described earlier [6]. Briefly, kidney tissue was washed with ice-cold phosphate buffer saline (PBS), cortex and medulla were separated. Tissue was weighed and homogenized with complete protease inhibitor cocktail. The resulting homogenates were centrifuged and the supernatant stored at − 80 °C until assayed.

### Measurement of pro-inflammatory cytokines

Camel ELISA kits (MyBiosource) were used to measure levels of IL-1β, IL-6 and IL-18 in kidney homogenate as per the manufacturer’s protocol. Briefly, sample and standards were added to the coated 96 well micro titer plate for 1 h at 37 °C. The absorbance was read at 450 nm, and expressed as pg/mg tissue [6].

### Estimation of oxidative stress biomarkers (GSH, CAT, SOD, and MDA)

Levels in kidney homogenate measured according to manufacturer’s protocol using commercially available kits [6]. Briefly, using a kinetic assay, GSH was reduced to glutathione disulfide (GSSG) and the product measured spectrophotometrically. For CAT assay the method was based on reaction of the enzyme with methanol in the presence hydrogen peroxide (H2O2) and d the product was measured spectrophotometrically. For SOD assay, we utilized the tetrazolium salt for the detection of superoxide radicals generated by xanthine oxidase and hypoxanthine. For MDA assay, we used the reaction of MDA with thiobarbituric acid (TBA) to form a MDA-TBA adduct that was absorbed strongly at 532 nm [6].

### Measurement of apoptotic markers

ELISAs and assays for caspases 3, 8, 9, BCL-xL, p53 and PARP1 were performed according to the manufacturer’s protocol as described earlier [6]. The absorbance was read with an Emax Plus microplate reader (Molecular devices, CA 94,089, USA). Results were expressed as ng/mg tissue.

### RNA extraction and cDNA synthesis

Frozen renal cortex and medulla were separated using sterile scalpel and a surgical saw. Tissue samples were crushed to a fine powder under liquid nitrogen with a mortar and a pestle. Powdered tissue was placed into 1.5 mL RNAse free tubes maintained on dry ice. Total RNA was extracted from approximately 30 mg of powdered tissue using a Direct-zol™ RNA MiniPrep kit (Zymo research; R2052) following the manufacturer’s instructions. For cDNA synthesis, 1000 ng of RNA from each sample was reversely transcribed using the GOScriptTM cDNA synthesis system (Promega; A276A).

### Real-time quantitative PCR analysis

Primers Table 3 were designed based on National Center for Biotechnology Information (NCBI) reference sequences (RefSeq) for target genes and were synthesized by Sigma-Aldrich®. The optimization and validation of primers were performed according to normal ABI protocols and the relative standard curve method [45]. The cDNA samples from reverse transcription were used as templates for the qPCR which was conducted in duplicates in 10 µl reaction volumes using Power UpTM SYBR Green Master Mix (Thermo Fisher Scientific,100,029,283) on an ABI StepOne-Plus Real-Time PCR System. The reference gene PPIA was used as the internal control gene for qPCR on the basis of its stability and robust CT value [24]. 2^−ΔΔCT^ method was applied for the relative quantification of gene expression [32]. Studies were performed in accord with MIQE guidelines [14].

**Table 3:** Primers for qPCR

Gene	Corresponding protein	NCBI reference sequence (mRNA)	5′ to 3′ primer sequence (F: forward, R: reverse)	Product size (bp)
*PPIA*	Peptidylprolyl isomerase A	XM_010987886.1	F: ACCACCAGACCATTCCTTCT R: TATGGAACCCCGAAAACTGC	109
*CAT*	Catalase	XM_011000575.1	F: AGACTTGCCCAGGAAGATCC R: GTCCAGGAGGGATAGTTGCC	80
*SOD1*	Superoxide dismutase 1	XM_010977357.1	F: ACCATCCACTTCGAGCAGAA R: ATGGACCCCGATACCATGAC	86
*SOD2*	Superoxide dismutase 2	NM_001319878.1	F: TACTGGAAGCCATCAACCGT R: GCAATCTGTAAGCGTCCCTG	136
*SOD3*	Superoxide dismutase 3	XM_010991051.1	F: CTCTGTGCCTACCTGCTCAT R: TGCCAGATCTCCGTCACTTT	128
*GCLC*	Glutamate- cysteine ligase catalytic subunit	XM_010992378.1	F: ACCAGAGTACGGGAGCTACA R: GTCCTCCACGGTGTTGAACT	85
*GSS*	Glutathione synthetase	XM_010975159.1	F: AGCTATGCCCCATTCACACT R: ACCAGCAGGTTGAAGTCCAT	92
*TBXAS1*	Thromboxane A synthase 1	XM_010983188.1	F: GCCCTGTTAGTGGTCCTCTT R: GGAGAAGGCTTGGGATGTCT	98
*PTGS1*	Prostaglandin- endoperoxide synthase 1	XM_010997991.1	F: CGTGGAGTTCAACCAGCTCTA R: AGGTGTTGAAGAGGAACTGCT	101
*PTGS2*	Prostaglandin- endoperoxide synthase 2	XM_010992100.1	F: CTGGTCTGATGATGTACGCC R: ACAACCGCTCATCATCCCAT	99
*ALDH1L1*	Aldehyde dehydrogenase 1 family member L1	XM_010998232.1	F: GTCCAGGGGTAGTCACCAAA R: CGTTCTTCACCAGCAGCATT	72
*ALDH1A1*	Aldehyde dehydrogenase 1 family member A1	XM_010999961.1	F: TCCTTTGGAAGATAGCGCCT R: GTCCGTAGCCTGGGACAATA	153
*ACSS1*	Acyl-CoA synthetase short	XM_010992742.1	F: GAGCTGAAGAGGATCGTGGAT R: TGCTCGAGGGGTATGTCCA	119
	chain family member 1			
*ACSS2*	Acyl-CoA synthetase short chain family member 2	XM_010975158.1	F: ACAGTTGGAGGCTACATGCT R: TGCACCAGAACACATCCTCT	82
*GPI*	Glucose-6-phosphate isomerase	XM_010989123.1	F: TGCCATTTTTCTGCAGCTTGA R: ATCACAGCCTCCGTCACAA	88
*IL-1B*	Interleukin 1 beta	XM_010984994.1	F: TGAACCCGCCAGTGAAATGA R: GACGCAGCACTTCATCTGTT	94
*IL-6*	Interleukin 6	XM_010987177.1	F: GGTACCCCTGGGAGAAGATT R: GCAGAGATTCTGCCGAGGAT	108
*IL-18*	Interleukin 18	XM_010986146.1	F: AGGTGTGGCAGTAACCATCT R: TCAGGAGGGCTCATTTCCTT	96
*CASP3*	Caspase 3	XM_010974562.1	F: GCTTCTTCAGAGGGGACAGT R: TCGGCAGGCCTGAATAATGA	71
*CASP8*	Caspase 8	XM_010991975.1	F: TGAAAGCAGTCCAGGGGAAA R: ATGTCTCTGGCTCACTGTCC	119
*CASP9*	Caspase 9	XM_011000878.1	F: CGGACGCCATGTCTAGTTTG R: TCCCTCCAGGAGACAAAACC	82
*TP53*	Tumor protein p53	XM_010996514.1	F: GCCCCTCCTCAGCATCTTAT R: CCACCACACTGTGTCGAAAA	88
*BCL2L1*	B-cell lymphoma-extra large	XM_010993888.1	F: GTTTGAACTGAGGTACCGGC R: CCCATCCCGGAAGAGTTCAT	115
*PARP1*	Poly ADP-ribose polymerase 1	XM_010992461.1	F: TGCATGGTCAAGACCCAGAT R: GGGAATATACGGTCCTGCCT	113

### Statistical Analysis

Statistical analyses were performed by independent t-test using SPSS (Statistical Package for Social Sciences, version 23: SPSS, Chicago, USA) or Graphpad Prism 8 software. Data shown as mean ± SEM and *p* value less than 0.05 defined statistically significant.

## Data Availability

The datasets used and/or analysed during the current study are available from the corresponding author on reasonable request.

## Abbreviations

BcL-xl: B-cell lymphoma-extra-large,
CAT: Catalase
DNA: Deoxyribonucleic acid
ELISA: Enzyme-linked immunosorbent assay
GSH: Total Glutathione
IL-1β: Interleukin 1β
IL-6: Interleukin 6
IL-18: Interleukin 18
MDA: Malondialdehyde
mRNA: Messenger Ribonucleic Acid
NCB1: National Center for Biotechnology Information
PARP1: Poly ADP-ribose Polymerase
p53: Tumor protein 53
qPCR: Quantitative Polymerase Chain Reaction
RNA: Ribonucleic acid
ROS: Reactive oxygen species
SOD: Superoxide dismutase
TNF-α: Tumor Necrosis Factor α
*SOD1, 2, 3*: Superoxide dismutase 1, 2, 3 genes
*TBXAS1*: Thromboxane A synthase 1
*PTGS1* and *PTGS2*: Prostaglandin-endoperoxide synthase 1 and 2
*ALDH1L1*: Formyl tetrahydrofolate dehydrogenase-10
*ALDH1A1*: Aldehyde Dehydrogenase 1 Family Member A1
*ACSS1*: Acyl-CoA Synthetase Short Chain Family Member 1
*ACSS2*: Acyl-CoA Synthetase Short Chain Family Member 2
GPI: Genuine progress indicator
*GCLC*: Glutamate-Cysteine Ligase Catalytic Subunit
*GSS*: Glutathione synthetase (GSH synthesis)
CASP3: Caspase 3
CASP8: Caspase 8
CASP9: Caspase 9
TP53: Tumor protein 53
*BCL2L1*: Is a human gene, it encodes both of the human proteins Bcl-xL and Bcl-xS
